# Supplementary figures and images for: Modelling the visual world of a velvet worm
Source: PLoS Comput Biol. 2021 Jul 28;17(7):e1008808. doi: 10.1371/journal.pcbi.1008808 (PMC8363015; doi:10.1371/journal.pcbi.1008808)

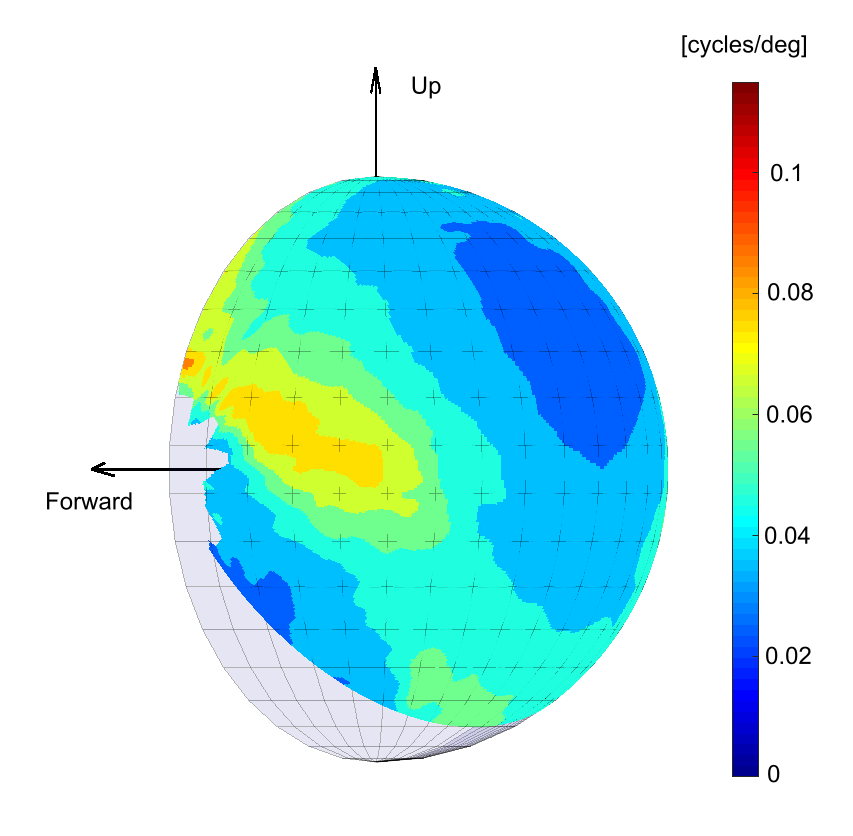

Supplement: S1 Fig — Computed resolution (spatial cut-off frequency) for horizontal structures using a 3D model with minimal altering after segmentation reconstruction. The cut off frequencies in the forward direction ~0.08 cycles/deg does not differ greatly from the optimized model ~0.1 cycles/deg. The cut-off frequency [cycles/deg] was determined by Fourier transform of the individual photoreceptor sensitivity functions and determining the FWHM of the response in the horizontal direction in the Fourier plane. (TIF) [file pcbi.1008808.s001.tif]

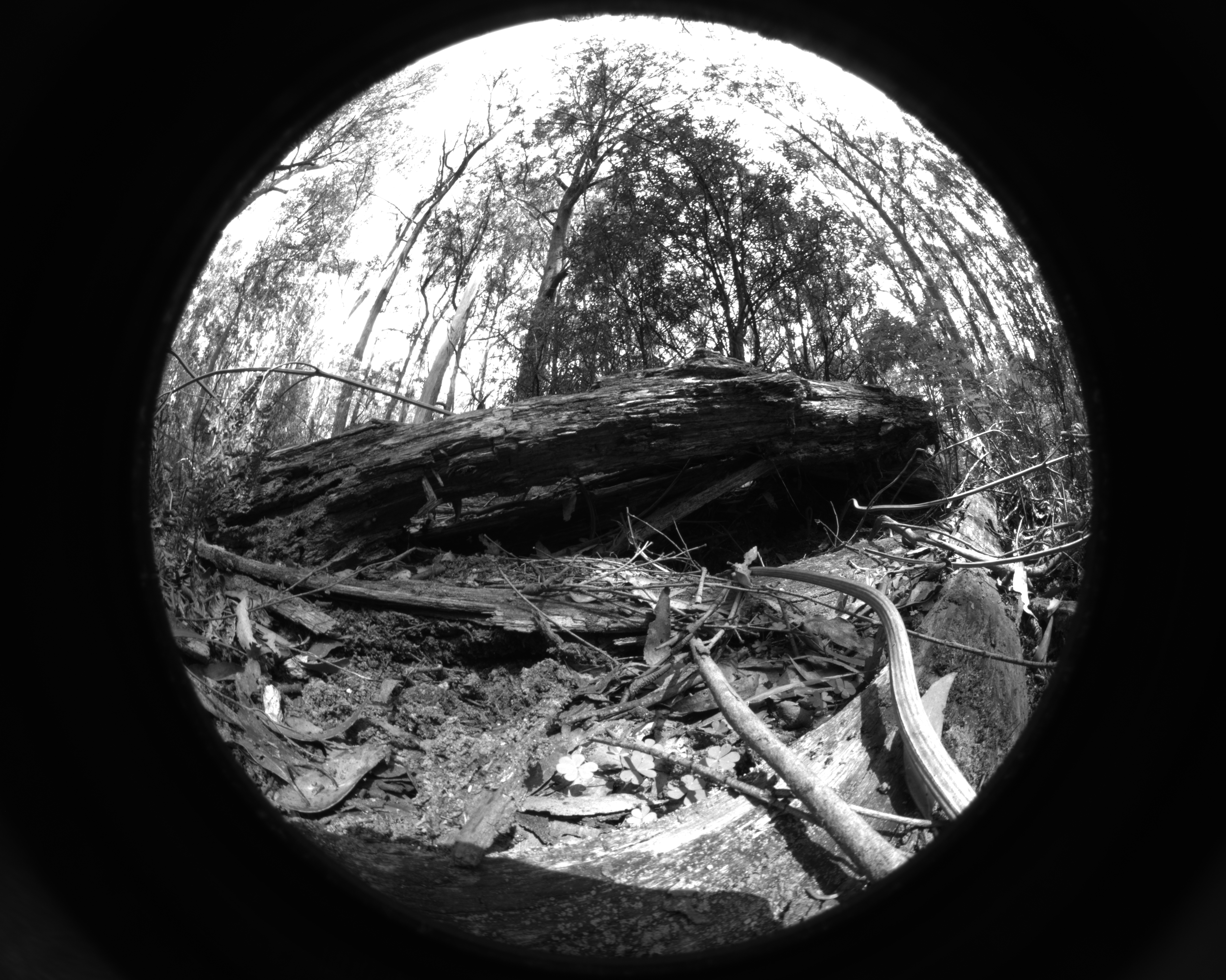

Supplement: S1 Code — The complete code used for raytracing and filter creation. This includes functions for importation and manipulation of surfaces and volumes, spatial partitioning, creation of photoreceptor approximation, ray source creation, raytracing, absorption calculation, absorption analysis, absorption result visualization, image filter creation and image filtering. Source code can be found at https://github.com/mLjungholm/Raytrace.git. (ZIP) [file pcbi.1008808.s002.zip › code/data/test_data/images_for_filter/log1.png]

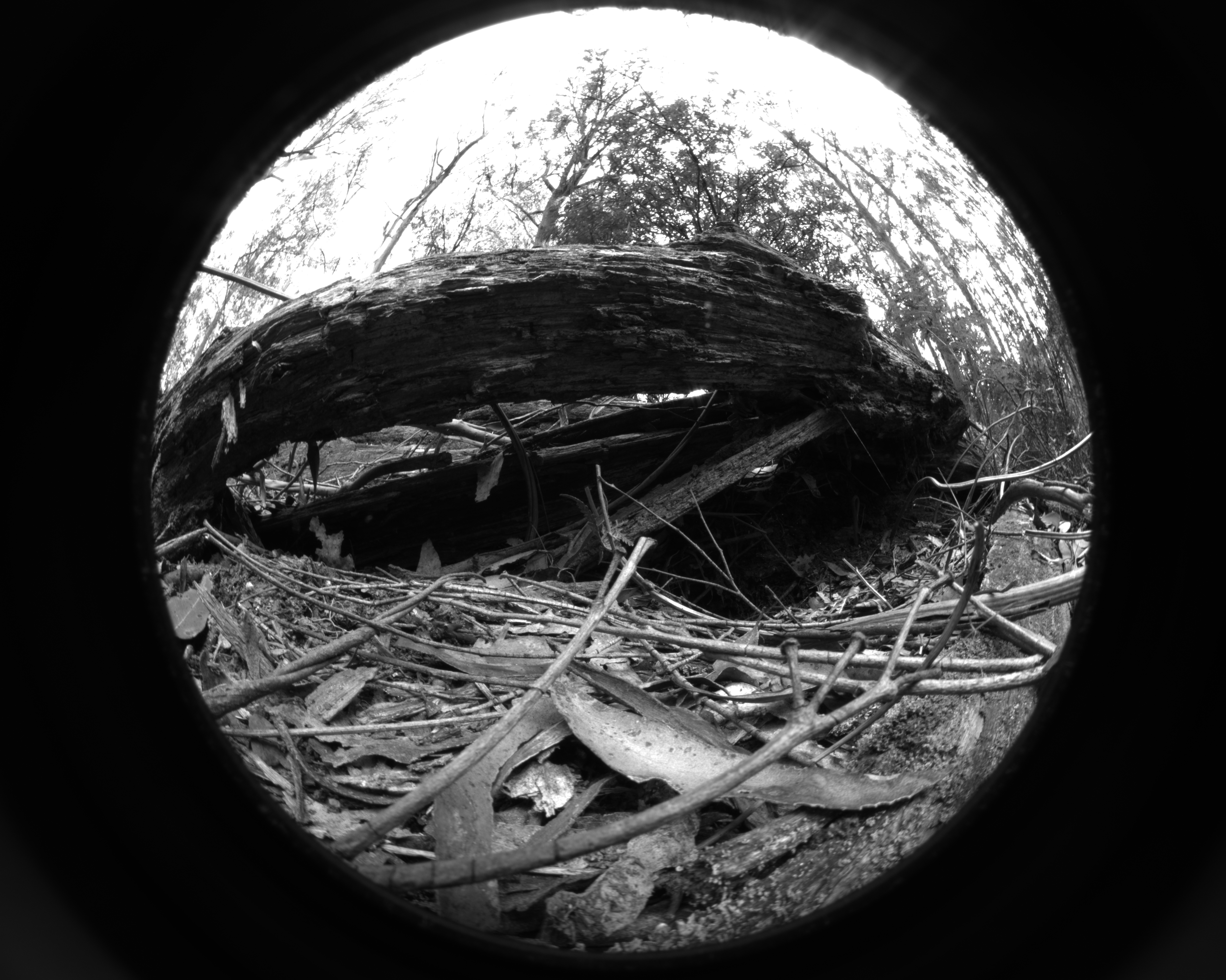

Supplement: S1 Code — The complete code used for raytracing and filter creation. This includes functions for importation and manipulation of surfaces and volumes, spatial partitioning, creation of photoreceptor approximation, ray source creation, raytracing, absorption calculation, absorption analysis, absorption result visualization, image filter creation and image filtering. Source code can be found at https://github.com/mLjungholm/Raytrace.git. (ZIP) [file pcbi.1008808.s002.zip › code/data/test_data/images_for_filter/log2.tif]

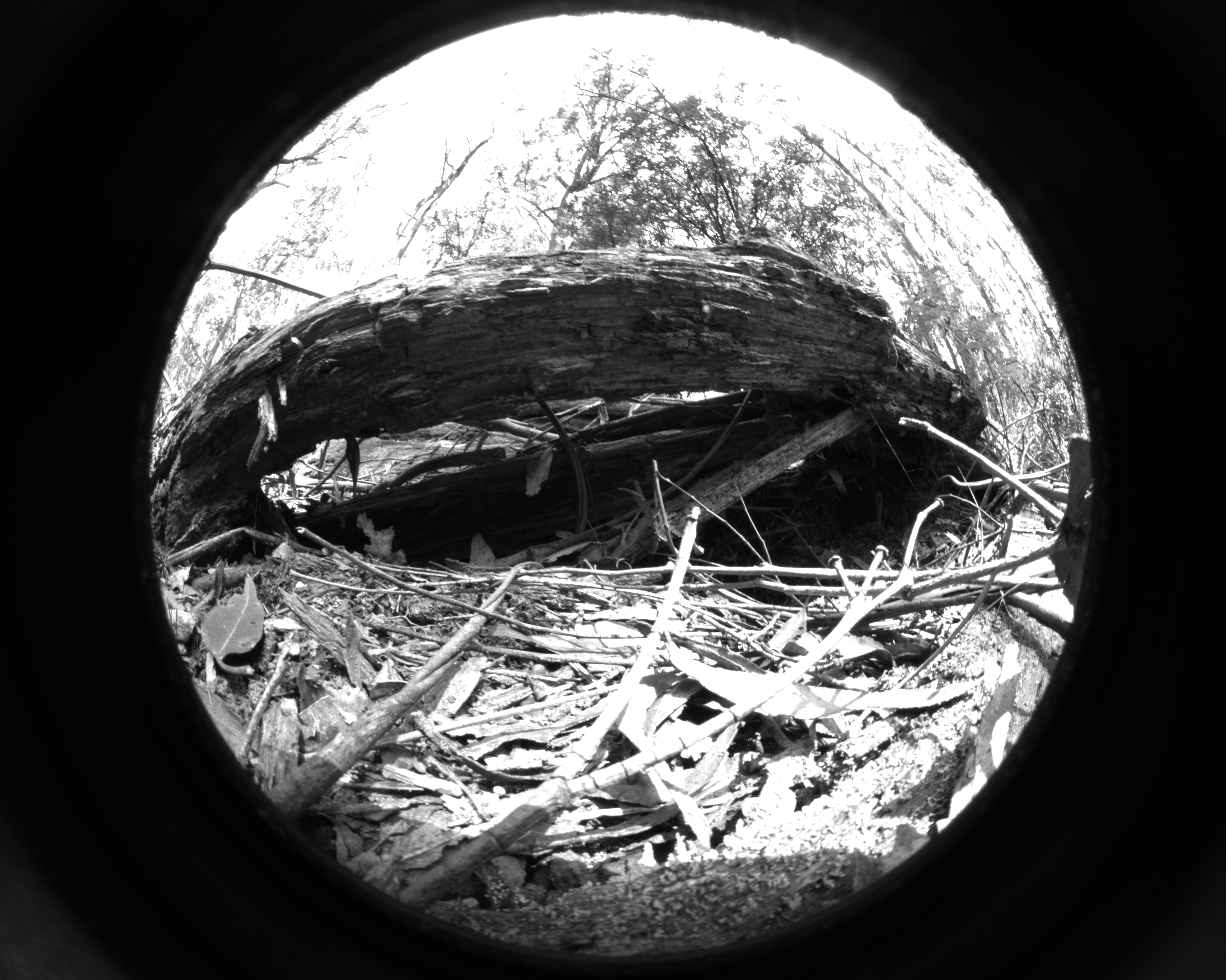

Supplement: S1 Code — The complete code used for raytracing and filter creation. This includes functions for importation and manipulation of surfaces and volumes, spatial partitioning, creation of photoreceptor approximation, ray source creation, raytracing, absorption calculation, absorption analysis, absorption result visualization, image filter creation and image filtering. Source code can be found at https://github.com/mLjungholm/Raytrace.git. (ZIP) [file pcbi.1008808.s002.zip › code/data/test_data/images_for_filter/log22.tif]

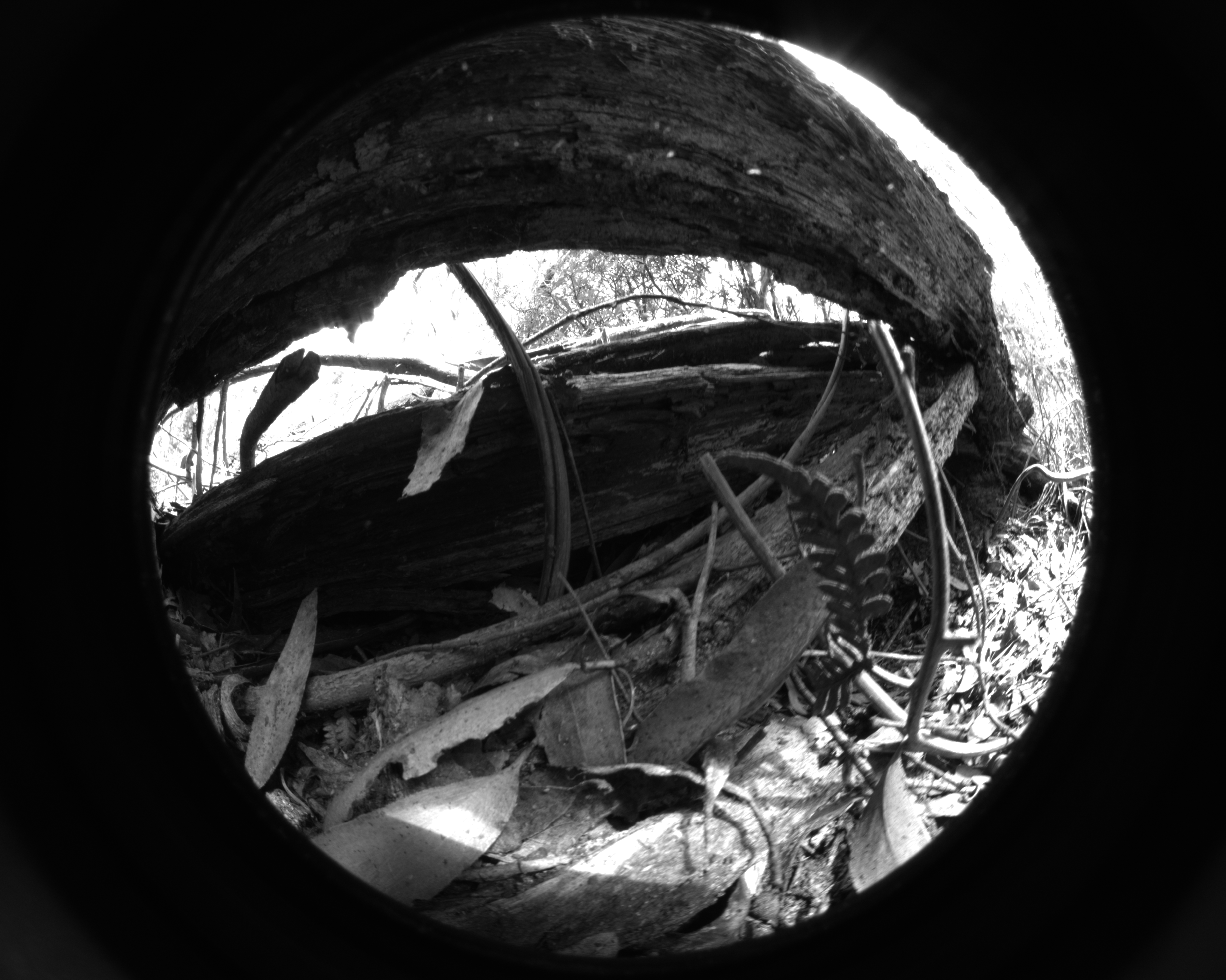

Supplement: S1 Code — The complete code used for raytracing and filter creation. This includes functions for importation and manipulation of surfaces and volumes, spatial partitioning, creation of photoreceptor approximation, ray source creation, raytracing, absorption calculation, absorption analysis, absorption result visualization, image filter creation and image filtering. Source code can be found at https://github.com/mLjungholm/Raytrace.git. (ZIP) [file pcbi.1008808.s002.zip › code/data/test_data/images_for_filter/log3.tif]

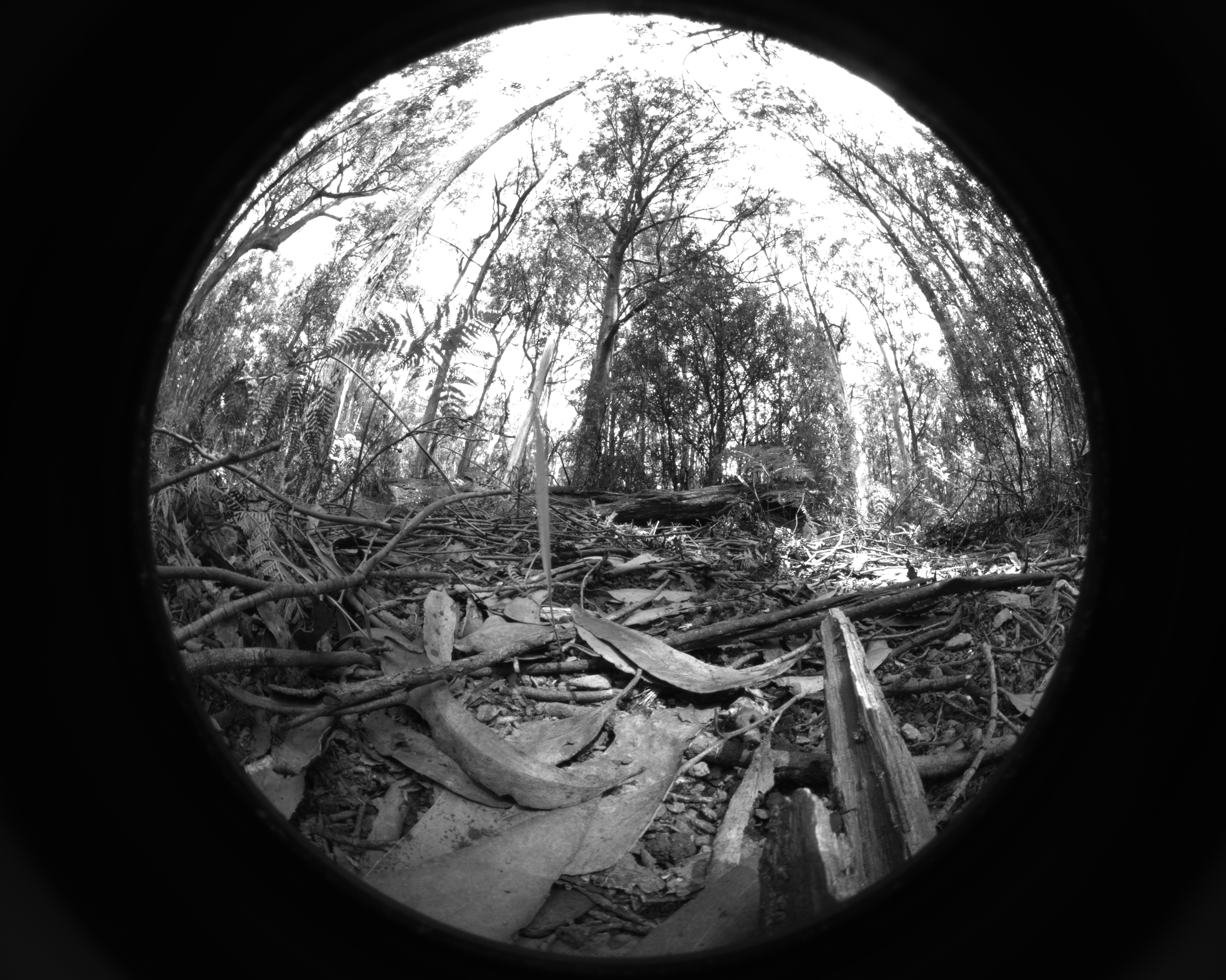

Supplement: S1 Code — The complete code used for raytracing and filter creation. This includes functions for importation and manipulation of surfaces and volumes, spatial partitioning, creation of photoreceptor approximation, ray source creation, raytracing, absorption calculation, absorption analysis, absorption result visualization, image filter creation and image filtering. Source code can be found at https://github.com/mLjungholm/Raytrace.git. (ZIP) [file pcbi.1008808.s002.zip › code/data/test_data/images_for_filter/log4.tif]

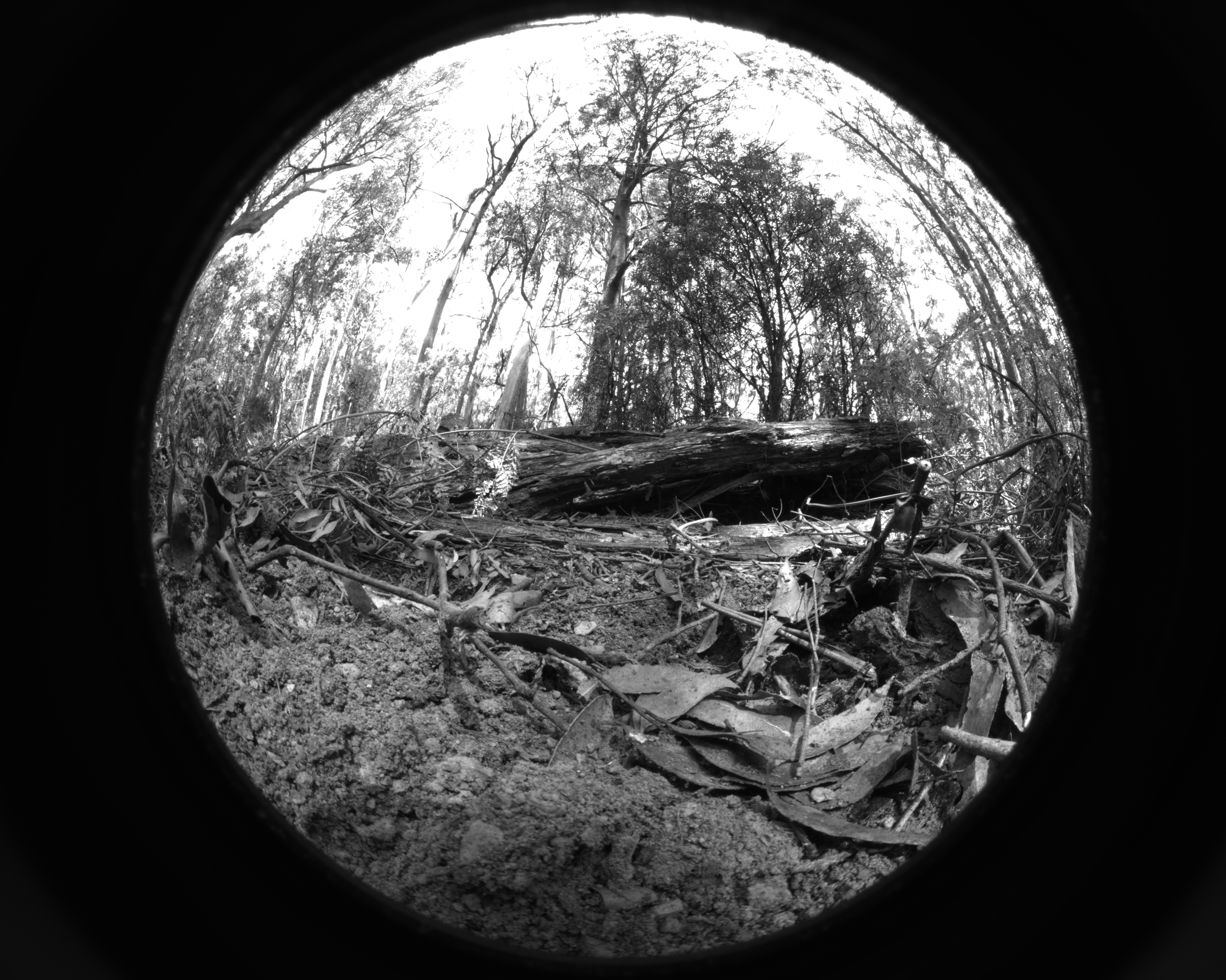

Supplement: S1 Code — The complete code used for raytracing and filter creation. This includes functions for importation and manipulation of surfaces and volumes, spatial partitioning, creation of photoreceptor approximation, ray source creation, raytracing, absorption calculation, absorption analysis, absorption result visualization, image filter creation and image filtering. Source code can be found at https://github.com/mLjungholm/Raytrace.git. (ZIP) [file pcbi.1008808.s002.zip › code/data/test_data/images_for_filter/log5.tif]

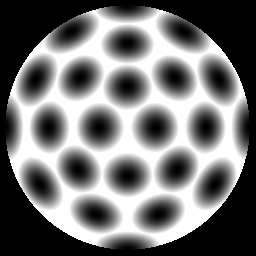

Supplement: S1 Code — The complete code used for raytracing and filter creation. This includes functions for importation and manipulation of surfaces and volumes, spatial partitioning, creation of photoreceptor approximation, ray source creation, raytracing, absorption calculation, absorption analysis, absorption result visualization, image filter creation and image filtering. Source code can be found at https://github.com/mLjungholm/Raytrace.git. (ZIP) [file pcbi.1008808.s002.zip › code/data/test_data/stimuli_images/cosine0.032.tiff]

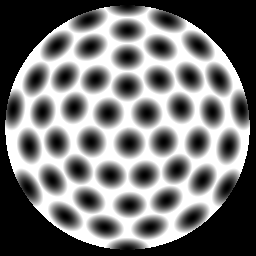

Supplement: S1 Code — The complete code used for raytracing and filter creation. This includes functions for importation and manipulation of surfaces and volumes, spatial partitioning, creation of photoreceptor approximation, ray source creation, raytracing, absorption calculation, absorption analysis, absorption result visualization, image filter creation and image filtering. Source code can be found at https://github.com/mLjungholm/Raytrace.git. (ZIP) [file pcbi.1008808.s002.zip › code/data/test_data/stimuli_images/cosine0.047.tiff]

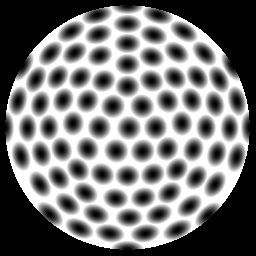

Supplement: S1 Code — The complete code used for raytracing and filter creation. This includes functions for importation and manipulation of surfaces and volumes, spatial partitioning, creation of photoreceptor approximation, ray source creation, raytracing, absorption calculation, absorption analysis, absorption result visualization, image filter creation and image filtering. Source code can be found at https://github.com/mLjungholm/Raytrace.git. (ZIP) [file pcbi.1008808.s002.zip › code/data/test_data/stimuli_images/cosine0.063.tiff]

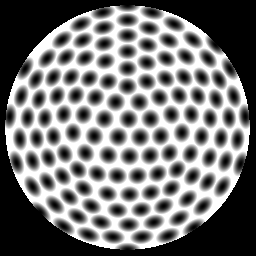

Supplement: S1 Code — The complete code used for raytracing and filter creation. This includes functions for importation and manipulation of surfaces and volumes, spatial partitioning, creation of photoreceptor approximation, ray source creation, raytracing, absorption calculation, absorption analysis, absorption result visualization, image filter creation and image filtering. Source code can be found at https://github.com/mLjungholm/Raytrace.git. (ZIP) [file pcbi.1008808.s002.zip › code/data/test_data/stimuli_images/cosine0.079.tiff]

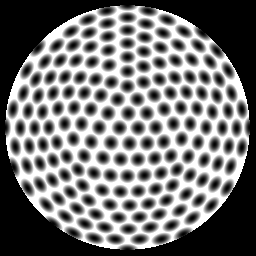

Supplement: S1 Code — The complete code used for raytracing and filter creation. This includes functions for importation and manipulation of surfaces and volumes, spatial partitioning, creation of photoreceptor approximation, ray source creation, raytracing, absorption calculation, absorption analysis, absorption result visualization, image filter creation and image filtering. Source code can be found at https://github.com/mLjungholm/Raytrace.git. (ZIP) [file pcbi.1008808.s002.zip › code/data/test_data/stimuli_images/cosine0.095.tiff]

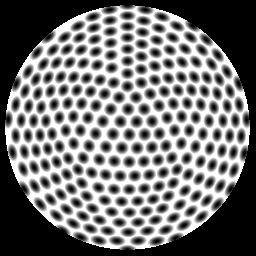

Supplement: S1 Code — The complete code used for raytracing and filter creation. This includes functions for importation and manipulation of surfaces and volumes, spatial partitioning, creation of photoreceptor approximation, ray source creation, raytracing, absorption calculation, absorption analysis, absorption result visualization, image filter creation and image filtering. Source code can be found at https://github.com/mLjungholm/Raytrace.git. (ZIP) [file pcbi.1008808.s002.zip › code/data/test_data/stimuli_images/cosine0.110.tiff]

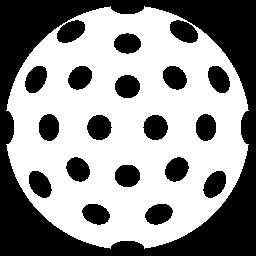

Supplement: S1 Code — The complete code used for raytracing and filter creation. This includes functions for importation and manipulation of surfaces and volumes, spatial partitioning, creation of photoreceptor approximation, ray source creation, raytracing, absorption calculation, absorption analysis, absorption result visualization, image filter creation and image filtering. Source code can be found at https://github.com/mLjungholm/Raytrace.git. (ZIP) [file pcbi.1008808.s002.zip › code/data/test_data/stimuli_images/tophat0.032.tiff]

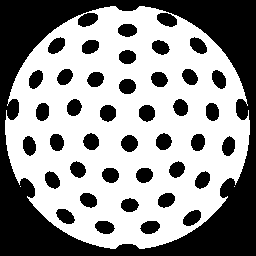

Supplement: S1 Code — The complete code used for raytracing and filter creation. This includes functions for importation and manipulation of surfaces and volumes, spatial partitioning, creation of photoreceptor approximation, ray source creation, raytracing, absorption calculation, absorption analysis, absorption result visualization, image filter creation and image filtering. Source code can be found at https://github.com/mLjungholm/Raytrace.git. (ZIP) [file pcbi.1008808.s002.zip › code/data/test_data/stimuli_images/tophat0.047.tiff]

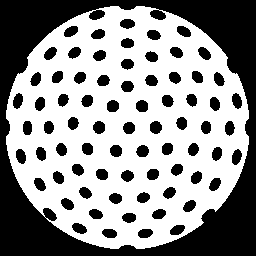

Supplement: S1 Code — The complete code used for raytracing and filter creation. This includes functions for importation and manipulation of surfaces and volumes, spatial partitioning, creation of photoreceptor approximation, ray source creation, raytracing, absorption calculation, absorption analysis, absorption result visualization, image filter creation and image filtering. Source code can be found at https://github.com/mLjungholm/Raytrace.git. (ZIP) [file pcbi.1008808.s002.zip › code/data/test_data/stimuli_images/tophat0.063.tiff]

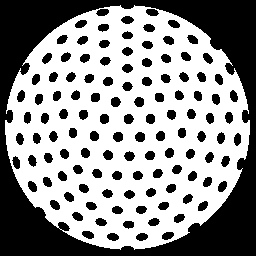

Supplement: S1 Code — The complete code used for raytracing and filter creation. This includes functions for importation and manipulation of surfaces and volumes, spatial partitioning, creation of photoreceptor approximation, ray source creation, raytracing, absorption calculation, absorption analysis, absorption result visualization, image filter creation and image filtering. Source code can be found at https://github.com/mLjungholm/Raytrace.git. (ZIP) [file pcbi.1008808.s002.zip › code/data/test_data/stimuli_images/tophat0.079.tiff]

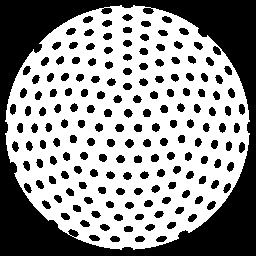

Supplement: S1 Code — The complete code used for raytracing and filter creation. This includes functions for importation and manipulation of surfaces and volumes, spatial partitioning, creation of photoreceptor approximation, ray source creation, raytracing, absorption calculation, absorption analysis, absorption result visualization, image filter creation and image filtering. Source code can be found at https://github.com/mLjungholm/Raytrace.git. (ZIP) [file pcbi.1008808.s002.zip › code/data/test_data/stimuli_images/tophat0.095.tiff]

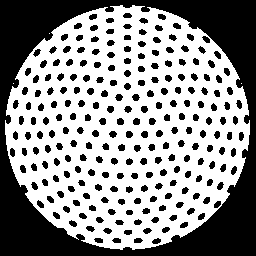

Supplement: S1 Code — The complete code used for raytracing and filter creation. This includes functions for importation and manipulation of surfaces and volumes, spatial partitioning, creation of photoreceptor approximation, ray source creation, raytracing, absorption calculation, absorption analysis, absorption result visualization, image filter creation and image filtering. Source code can be found at https://github.com/mLjungholm/Raytrace.git. (ZIP) [file pcbi.1008808.s002.zip › code/data/test_data/stimuli_images/tophat0.110.tiff]

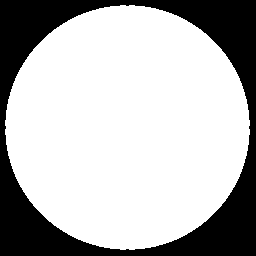

Supplement: S1 Code — The complete code used for raytracing and filter creation. This includes functions for importation and manipulation of surfaces and volumes, spatial partitioning, creation of photoreceptor approximation, ray source creation, raytracing, absorption calculation, absorption analysis, absorption result visualization, image filter creation and image filtering. Source code can be found at https://github.com/mLjungholm/Raytrace.git. (ZIP) [file pcbi.1008808.s002.zip › code/data/test_data/stimuli_images/white.tif]

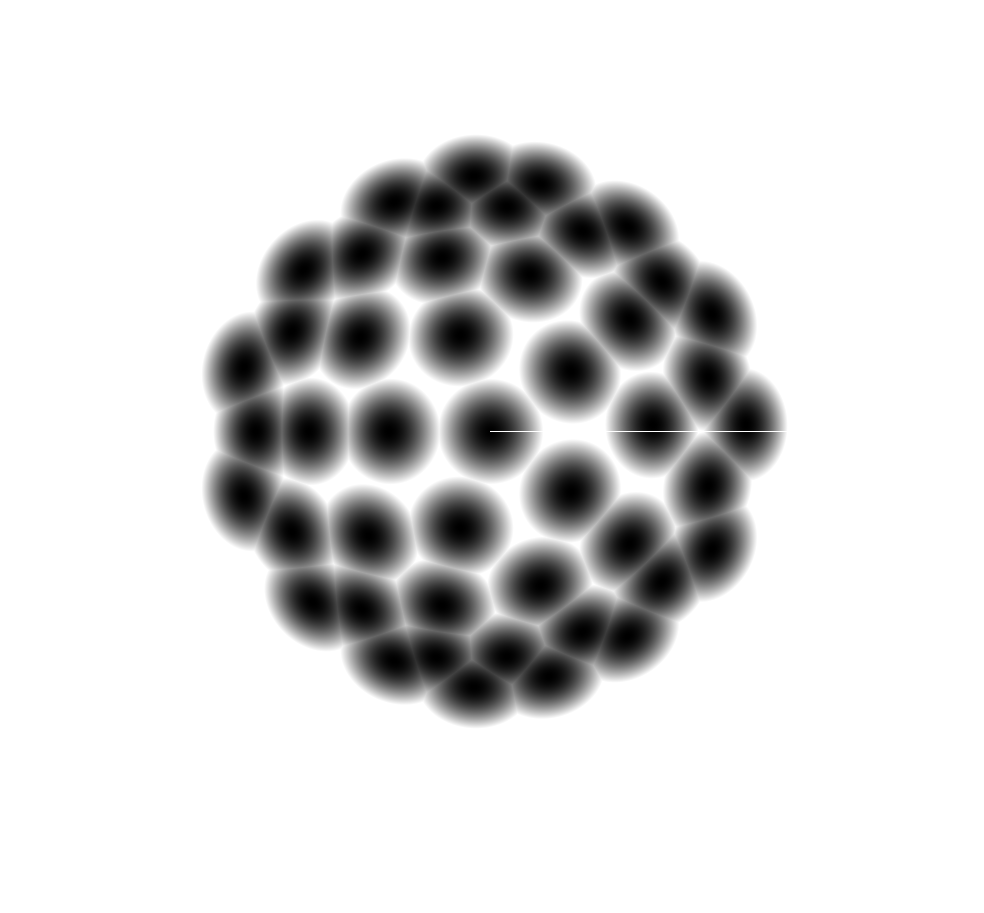

Supplement: S1 Code — The complete code used for raytracing and filter creation. This includes functions for importation and manipulation of surfaces and volumes, spatial partitioning, creation of photoreceptor approximation, ray source creation, raytracing, absorption calculation, absorption analysis, absorption result visualization, image filter creation and image filtering. Source code can be found at https://github.com/mLjungholm/Raytrace.git. (ZIP) [file pcbi.1008808.s002.zip › code/stimuli/hexagon_cosine_21.3deg.png]

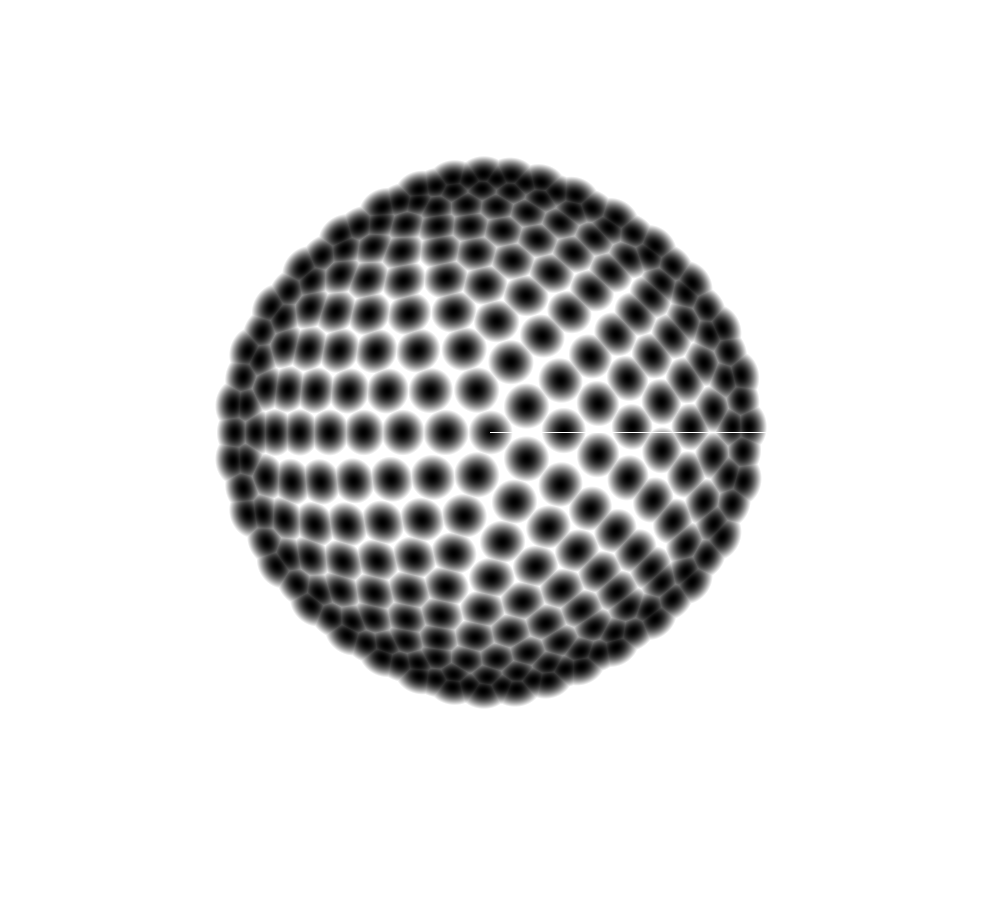

Supplement: S1 Code — The complete code used for raytracing and filter creation. This includes functions for importation and manipulation of surfaces and volumes, spatial partitioning, creation of photoreceptor approximation, ray source creation, raytracing, absorption calculation, absorption analysis, absorption result visualization, image filter creation and image filtering. Source code can be found at https://github.com/mLjungholm/Raytrace.git. (ZIP) [file pcbi.1008808.s002.zip › code/stimuli/hexagon_cosine_9.2deg.png]

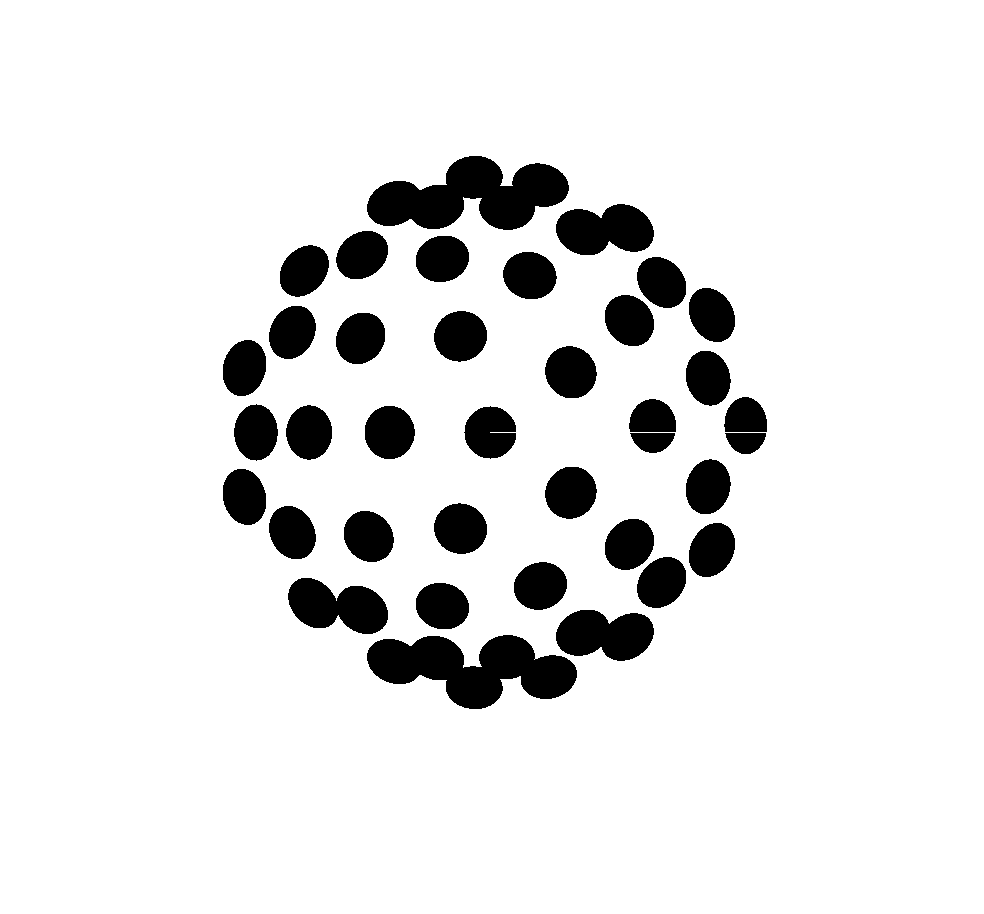

Supplement: S1 Code — The complete code used for raytracing and filter creation. This includes functions for importation and manipulation of surfaces and volumes, spatial partitioning, creation of photoreceptor approximation, ray source creation, raytracing, absorption calculation, absorption analysis, absorption result visualization, image filter creation and image filtering. Source code can be found at https://github.com/mLjungholm/Raytrace.git. (ZIP) [file pcbi.1008808.s002.zip › code/stimuli/hexagon_tophat_21.3deg.png]
