# Supplementary material for: Modelling the visual world of a velvet worm
Source: PLoS Comput Biol. 2021 Jul 28;17(7):e1008808. doi: 10.1371/journal.pcbi.1008808 (PMC8363015; doi:10.1371/journal.pcbi.1008808)
Supplement: Attachment — Submitted filename: Response to Reviewers.docx [file pcbi.1008808.s003.docx]

We much appreciate the kind and constructive comments on our paper.

All the minor comments (typos and similar errors) have been corrected as suggested by referees 1, 2 and 3. We are sorry for the large number of typos, noted by referee 1, and have now carefully gone through the manuscript. In fact, referee 2 listed most of these errors.

Some of the comments and suggestions required a little more than just correcting a typo or changing a word, and these changes are listed below in red after the referee’s comments.

Referee 1:

It may be worthwhile to reiterate that the lens is assumed to be homogeneous, as indicated by the previous optical studies.

This is now added at the top of page 4

And, a definition of what is considered to be a rhabdom vs rhabdomeres (line 103) may be worthwhile.

To avoid confusion, we have now consistently changed all ”rhabdomeres” to ”rhabdoms”. The distinction does not have much meaning in these retinas.

One example of other sloppiness: in Fig. 2, pg is pigment granules, in Fig. 4 screening pigment layer.

We have now consistently changed this to ”screening-pigment layer”.

Fig. 4 has no A and B

We have added A and B to the figure.

Referee 2:

Although covered nicely in the discussion, the introduction could do with more information on the behaviour and ecology of the velvet worm, and the various visual tasks that they are known to perform.

We have now added some information to the last paragraph of the Introduction.

Please explain why the internal area of the eye is classified as a lens rather than an empty void of vitreous. Is it a hard structure?

This is now explained at the top of page 4

The selection of the ‘best eye’ (line 125) was somewhat subjective. Is there a way you can make this selection process more robust? Are you confident that all the eyes were not subjected to the same fixation artefacts, causing similar deformation across all eyes, and therefore also in your ‘best eye’? Any additional information that improves the readers confidence in this eye selection process would be welcome.

We agree that this needed a better explanation. We have thus modified the text in the 3^rd^ paragraph of the Results to explain that the general asymmetric shape of the eye was very consistent between eyes, and also seen clearly during dissection of the eyes before fixation. The randomly occurring dimples of the pigment layer, as seen in the sections, was not seen during dissection before fixation, and were thus interpreted as artifacts of fixation and embedding, probably caused by different amounts of shrinkage of the retinal volume and the surrounding pigment layer. This is now explained in the text. We also mention that modelling with or without such artifacts produce essentially identical results.

Some of the figures would be better consolidated into multiple panels of a single figure. For example figure 1 and 2 would work well consolidated together. Also figures 5 and 6.

We agree and have joined the figures as suggested. The figure caption and figure numbers have been altered accordingly.

Figure 7 – Is ‘intensity’ the most appropriate label for the colourmap? This could easily cause confusion with light intensity.

We understand the problem and have changed the label and legend explanation to “receptor light-absorption”, which is a more correct description.

Figure 8 – The label ‘Relative light absorption’ is a little misleading. Is this a measure of retinal thickness? Why not use ‘retinal thickness as your label, given that yosorptiu are not directly measuring light absorption?

Our original label is actually correct. It is the computed light absorption mapped over the visual field, and not just retinal thickness. We have made this clearer in the legend.

Figure 9 – It would be great to have an indication on the figure or in the caption of the angular size of the image. Presumably that the main image is a flattened hemisphere with an angular size of 180 degrees.

We have added a clarification in the caption and the figure. A scalebar is problematic since the projection of the image results in a variable scale from the centre of the image.

Referee 3:

My only minor concern is that the eye morphology examined may not be as universal for the species as assumed. It is quite an asymmetric eye in an animal that does not appear to require high-resolution vision, so natural selection for exact eye shape may be quite relaxed. The authors do say they picked one eye out of ten as representative, but it would be worth seeing a panel of the other eyes to get a sense of variation.

This is similar to a point raised by referee 2, and we have modified the 3^rd^ paragraph of the Results, as explained in our comments to referee 2 above. As explained, the asymmetric retinal shape is very consistent and clearly not an artifact. It improves visual performance in the forward direction.

There may also be variation with respect to age, sex, and population, so I think the authors should be cautious about their conclusions.

In the last sentence of the Discussion, we now point out that there is no obvious sexual dimorphism, but that the smaller eyes of juveniles of course will not perform quite as well as the large adult eyes.
